# Maternal, obstetric, and fetal Doppler characteristics in a high-risk population: prediction of adverse perinatal outcomes and of cesarean section due to intrapartum fetal compromise

**DOI:** 10.1590/0100-3984.2022.0104

**Published:** 2023

**Authors:** Jonas de Lara Fracalozzi, Marcos Masaru Okido, Gerson Cláudio Crott, Geraldo Duarte, Ricardo de Carvalho Cavalli, Edward Araujo Júnior, Alberto Borges Peixoto, Alessandra Cristina Marcolin

**Affiliations:** 1 Department of Gynecology and Obstetrics, Faculdade de Medicina de Ribeirão Preto da Universidade de São Paulo (FMRP-USP), Ribeirão Preto, SP, Brazil; 2 Department of Obstetrics, Escola Paulista de Medicina da Universidade Federal de São Paulo (EPM-Unifesp), São Paulo, SP, Brazil; 3 Medical Course, Universidade Municipal de São Caetano do Sul (USCS), Campus Bela Vista, São Paulo, SP, Brazil; 4 Department of Obstetrics and Gynecology, Universidade Federal do Triângulo Mineiro (UFTM), Uberaba, MG, Brazil; 5 Gynecology and Obstetrics Service, Hospital Universitário Mário Palmério, Universidade de Uberaba (Uniube), Uberaba, MG, Brazil

**Keywords:** Ultrasonography, Doppler, Ultrasonography, prenatal, Fetal growth retardation/physiopathology, Cesarean section/statistics & numerical data, Middle cerebral artery/diagnostic imaging, Umbilical arteries/diagnostic imaging, Ultrassonografia Doppler, Ultrassonografia pré-natal, Retardo do crescimento fetal/fisiopatologia, Cesárea/estatística & dados numéricos, Artéria cerebral média/diagnóstico por imagem, Artérias umbilicais/diagnóstico por imagem

## Abstract

**Objective:**

To evaluate the capacity of fetal Doppler, maternal, and obstetric characteristics for the prediction of cesarean section due to intrapartum fetal compromise (IFC), a 5-min Apgar score < 7, and an adverse perinatal outcome (APO), in a high-risk population.

**Materials and Methods:**

This was a prospective cohort study involving 613 singleton pregnant women, admitted for labor induction or at the beginning of spontaneous labor, who underwent Doppler ultrasound within the last 72 h before delivery. The outcome measures were cesarean section due to IFC, a 5-min Apgar score < 7, and any APO.

**Results:**

We found that maternal characteristics were neither associated with nor predictors of an APO. Abnormal umbilical artery (UA) resistance index (RI) and the need for intrauterine resuscitation were found to be significant risk factors for cesarean section due to IFC (*p* = 0.03 and *p* < 0.0001, respectively). A UA RI > the 95th percentile and a cerebroplacental ratio (CPR) < 0.98 were also found to be predictors of cesarean section due to IFC. Gestational age and a UA RI > 0.84 were found to be predictors of a 5-min Apgar score < 7 for newborns at < 29 and ≥ 29 weeks, respectively. The UA RI and CPR presented moderate accuracy in predicting an APO, with areas under the ROC curve of 0.76 and 0.72, respectively.

**Conclusion:**

A high UA RI appears to be a significant predictor of an APO. The CPR seems to be predictive of cesarean section due to IFC and of an APO in late preterm and term newborns.

## Abstract (pt)

**Objetivo:**

Avaliar a capacidade do Doppler fetal e características materno-obstétricas na predição de cesariana por comprometimento fetal intraparto (CFI), índice de Apgar de 5º min < 7 e desfecho perinatal adverso (DPA) em uma população de alto risco.

**Materiais e Métodos:**

Estudo de coorte prospectivo envolvendo 613 parturientes admitidas para indução ou em início de trabalho de parto espontâneo que realizaram ultrassonografia Doppler nas 72 horas anteriores ao parto. Os desfechos foram cesariana por CFI, índice de Apgar de 5º min < 7 e DPA.

**Resultados:**

As características maternas não foram associadas nem preditoras de DPA. Índice de resistência (IR) da artéria umbilical (AU) anormal (*p* = 0,03) e necessidade de medidas de ressuscitação intrauterina (*p* < 0,0001) permaneceram como fatores de risco significativos para cesariana por CFI. IR AU > 95º e razão cerebroplacentária (RCP) < 0,98 foram preditores de cesariana. Idade gestacional e IR AU > 0,84 foram os preditores de índice de Apgar de 5º min < 7 para recém-nascidos < 29 e ≥ 29 semanas, respectivamente. IR AU e RCP apresentaram acurácia moderada na predição de DPA (área sob a curva ROC de 0,76 e 0,72, respectivamente).

**Conclusão:**

IR UA mostrou-se preditor significativo de DPA. RCP revelou-se possível preditora de cesariana por CFI e DPA em recémnascidos prematuros tardios e a termo.

## INTRODUCTION

Acute fetal compromise is one of the leading causes of perinatal morbidity and mortality worldwide. As well as being a major cause of fetal death, a reduction in the oxygen supply for the conceptus can lead to relevant neonatal complications^(1)^. Intrapartum fetal compromise (IFC) occurs in a significant proportion (10-15%) of cases of intrapartum hypoxia-ischemia^(2)^. In most cases, IFC occurs in low-risk pregnancies, making adverse perinatal outcomes (APOs) difficult to predict^(3)^.

Although electronic monitoring of the fetal heart rate (FHR), known as cardiotocography (CTG), is widely used in screening for IFC, its use has not reduced the incidence of hypoxic-ischemic encephalopathy, probably due to its low positive predictive value in diagnosing fetal hypoxia^(4,5)^. Technologies complementary to CTG were developed to reduce false-positive rates and avoid unnecessary interventions in cases of suspected fetal compromise, although there are still many uncertainties regarding the benefits of such technologies and technical difficulties related to their use, which hinder their introduction into clinical practice^(6-10)^. When acute fetal compromise becomes established, various physiological changes, including the redistribution of the fetoplacental blood flow, occur and behave as defense mechanisms; that is, attempts to maintain adequate oxygen levels in vital organs for fetal survival (the “brain sparing” effect). This phenomenon can be evidenced by cerebral blood flow Doppler^(11,12)^, which can show a reduction in the resistance index (RI) of the fetal middle cerebral artery (MCA).

Despite the seemingly beneficial role of the fetal brain sparing effect, some recent studies have produced results that call into question its protective effect, which can be greater or lesser depending on the gestational age (GA) at which it occurs and whether other hemodynamic parameters are normal or abnormal^(13-16)^. Studies have shown that, in cases of in late-onset fetal growth restriction (FGR), there is an association between the brain sparing effect and the occurrence of an APO^(17-19)^. However, this phenomenon is a late manifestation of fetal compromise, and, given its potentially harmful consequences, there is a need for methods that can detect signs of fetal blood flow redistribution earlier.

For normal and FGR fetuses, the cerebroplacental ratio (CPR) has emerged as predictor of cesarean section due to IFC, an APO, and long-term neurological impairment^(20-25)^. Because it represents the ratio between the RI of the MCA and that of the umbilical artery (UA), the CPR can be abnormal even before the brain sparing effect has been identified^(3)^. Nevertheless, studies evaluating the CPR and its association with the occurrence of an APO vary widely in terms of methodology and outcome measures. There is therefore insufficient evidence to recommend its introduction into clinical practice as a predictor of poor obstetric outcomes and neonatal complications.

The objective of this study was to evaluate the capacity of fetal Doppler, as well as of maternal and obstetric characteristics, to predict, in a high-risk population, cesarean section due to IFC, a 5-min Apgar score < 7, and the occurrence of an APO.

## MATERIALS AND METHODS

This was a prospective cohort study that included high-risk pregnant women admitted for labor induction due to maternal conditions or pregnant women admitted at the beginning of spontaneous labor (cervical dilation < 3.0 cm). All patients were recruited from the University Hospital of the Ribeirão Preto Medical School of University of São Paulo (FMRP-USP), in the city of Ribeirão Preto, Brazil, in 2018. The facility serves approximately 1,800 high-risk pregnant women every year. The inclusion criteria were GA > 26 weeks at delivery; singleton pregnancy; fetus without congenital anomalies; and obstetric ultrasound within the last 72 h before delivery. The following exclusion criteria were applied: structural or chromosomal anomalies diagnosed in the newborn; transfer of the newborn to another hospital; and failure to acquire data from the medical records. The study was approved by the FMRP-USP Research Ethics Committee (Reference no. 14366/2009). All participating patients gave written informed consent.

Of 1,167 eligible pregnant women, 554 did not meet the study criteria and were excluded, leaving 613 for analysis. The GA was determined on the basis of the last menstrual period and confirmed by a first trimester scan using the crown-rump length, or by the head circumference measured in a second trimester scan, performed between weeks 14 and 20 of pregnancy.

All ultrasound examinations performed before admission were conducted transabdominally with a 4-8 MHz probe (Voluson 730 Expert, GE Medical Systems, Milwaukee, WI, USA) by consultants with considerable experience in fetal medicine. The following parameters were measured: fetal biometry and estimated fetal weight (EFW), as described previously^(26)^; single deepest vertical pocket (SDVP) of amniotic fluid^(27)^; and the RIs of the UA and MCA on Doppler ultrasound. The UA was assessed at the level of umbilical cord insertion into the placenta, and the RI was considered abnormal if the value was above the 95th percentile^(28)^. The RI of the fetal MCA was assessed in a cross-sectional view of the fetal head at the level of the origin of the MCA from the circle of Willis and was considered abnormal if the value was below the 5th percentile^(29)^. Flow velocity waveforms were obtained without uterine contractions, fetal body movements, or maternal respiratory movements, when the FHR was normal. The UA and MCA were evaluated with color Doppler. The pulsed Doppler sample volume was < 3.0 mm, the insonation angle was < 30°, and the filter was set at < 50 Hz. The CPR was calculated by dividing the RI of the MCA by that of the UA, and the resulting value was considered abnormal if < 1.0^(30)^.

Intrapartum electronic FHR analysis was carried out in accordance with the National Institute of Child Health and Human Development guidelines^(31)^ and the American College of Obstetricians and Gynecologists (ACOG) guidelines^(32)^. If IFC was suspected, the pregnancies were managed in accordance with the ACOG guidelines^(6)^. Indeterminate (category II) or abnormal (category III) FHR tracings, even after 10-15 min of intrauterine resuscitation, corresponded to IFC.

Obstetric care was provided in accordance with the local guidelines, which are based on the best available evidence^(33)^. It is noteworthy that patients with absent or reversed end-diastolic velocity in the fetal UA or increased fetal ductus venosus pulsatility index undergo cesarean section at our institution. In addition, IFC was defined, according to International Federation of Gynecology and Obstetrics criteria^(34)^, as a fetal biophysical profile score ≤ 4 before delivery or a CTG result classified as pathological after delivery. However, the following were not considered indications for preterm delivery or cesarean section: a CPR or MCA RI < the 5th percentile; a UA RI > the 95th percentile with positive end-diastolic velocity; the fetus being categorized as small for gestational age (SGA), with an EFW < the 3rd percentile or < the 10th percentile plus abnormal Doppler findings; and GA < 37 weeks.

Variables of interest related to maternal characteristics, maternal history, ultrasound findings, intrapartum interventions, and newborn characteristics were obtained from each pregnant woman at enrollment in the study and from medical records. The following outcomes were considered: cesarean section due to IFC; a 5-min Apgar score < 7; and any APO. The occurrence of an APO was defined as one or more of the following: intraventricular hemorrhage; periventricular leukomalacia; hypoxic-ischemic encephalopathy; necrotizing enterocolitis; bronchopulmonary dysplasia; sepsis; admission of the newborn to the neonatal intensive care unit; and neonatal death. The local neonatology unit is a member of the Vermont Oxford Network and therefore applies the diagnostic criteria established by the network for each neonatal complication^(35)^.

### Statistical analysis

The sample calculation was performed using Proc Power procedure in the Statistical Analysis System, version 9.3 (SAS Institute Inc., Cary, NC, USA). Continuous variables are presented as mean and standard deviation, whereas categorical variables are expressed as absolute and relative frequencies. The chi-square test was employed to detect associations between qualitative variables and outcomes. A binomial log regression model (the Proc Logistic procedure in SAS, version 9.0; SAS Institute Inc.) was used in order to estimate the crude and adjusted relative risks (RRs) for each variable in relation to the outcomes. A conditional inference tree model was employed to determine the most significant predictors for the outcomes, which allowed the use of quantitative and qualitative variables, with R software, version 3.6.1. (The R Project for Statistical Computing, Vienna, Austria). Receiver operating characteristic (ROC) curves were constructed to evaluate the individual accuracy of each parameter for predicting outcomes by calculating the area under the curve (AUC). Values of *p* < 0.05 were considered statistically significant.

## RESULTS

The final study sample comprised 613 singleton pregnant women: 542 (88.4%) admitted for labor induction; and 71 (11.6%) admitted at the beginning of labor. The mean maternal age was 28.4 ± 6.97 years (range, 50-13 years), 319 (54.3%) of the pregnant women were obese, and the mean maternal weight was 90.3 ± 22.2 kg (range, 35-188 kg). The mean GA at delivery was 38 ± 3.2 weeks (range, 25-42 weeks), the mean birth weight was 2,914 ± 813 g (range, 560-5,030 g), and 179 (29.2%) of the newborns were classified as SGA. The mean RI for the UA and the MCA was 0.58 ± 0.10 (range, 0.36-1.02) and 0.73 ± 0.07 (range, 0.50-0.91), respectively. The mean CPR was 1.27 ± 0.27 (range, 0.36-1.02), and the mean SDVP was 3.91 ± 1.81 cm (range, 0.0-15.0 cm).

Tables 1 and 2 show the RRs for maternal, ultrasound, intrapartum, and neonatal variables in relation to cesarean section due to IFC, a 5-min Apgar score < 7, and any APO. None of the maternal characteristics were associated with any of those outcomes. Conversely, UA RI, MCA RI, CPR, SDVP, and prematurity (GA < 37 weeks) were associated with all three of the outcomes. Cesarean section, whether indicated or not, was associated with a 5-min Apgar score < 7 and with the occurrence of an APO. The occurrence of an APO and cesarean section due to IFC were both associated with an SGA neonate: the total cesarean section rate was 56.0% for SGA neonates, versus 35.0% for normal-birth-weight neonates; and the rate of cesarean section due to IFC was 31.8% for SGA neonates, compared with 13.0% for normal-birth-weight neonates (*p* = 0.004). During the study period, the overall rate of cesarean section in our high-risk neonatology unit was 42.0%.

**Table 1:** Univariate analysis of the associations of maternal, ultrasound, obstetric, and neonatal variables with cesarean section due to IFC.

Variable	Cesarean section		
	Yes n (%)	No n (%)	RR (95% CI)
Maternal age (years)			
≤ 19	9 (7.9)	61 (12.2)	0.70 (0.36-1.33)
19-35	75 (65.8)	335 (67.1)	Reference
≥ 35	30 (26.3)	103 (20.7)	1.23 (0.84-1.79)
Skin color			
White	72 (63.2)	328 (65.7)	Reference
Non-White	42 (36.8)	171 (34.3)	0.91 (0.64-1.28)
Tobacco use			
Yes	14 (12.3)	55 (11.0)	1.10 (0.66-1.81)
No	100 (87.7)	444 (89.0)	Reference
Chronic disease (maternal)			
Yes	95 (83.3)	381 (76.4)	1.43 (0.91-2.26)
No	19 (16.7)	118 (23.6)	Reference
Obesity			
Yes	58 (53.7)	261 (54.5)	0.97 (0.69-1.37)
No	50 (46.3)	218 (45.5)	Reference
Parity			
Primigravida	48 (42.1)	174 (34.9)	1.28 (0.91-1.78)
Multigravida	66 (57.9)	325 (65.1)	Reference
UA Doppler			
Normal	86 (75.4)	481 (96.4)	Reference
Abnormal	28 (24.6)	18 (3.6)	4.01 (2.96-5.43)
MCA Doppler			
Normal	77 (67.5)	420 (84.2)	Reference
Abnormal	37 (32.5)	79 (15.9)	2.05 (1.47-2.88)
CPR			
Normal	75 (65.8)	456 (91.4)	Reference
Abnormal	39 (34.2)	43 (8.6)	3.36 (2.47-4.58)
SDVP of amniotic fluid			
Normal	76 (66.7)	398 (79.8)	Reference
Abnormal	38 (33.3)	101 (20.2)	1.70 (1.21-2.40)
GA at delivery			
Term (≥ 37 weeks)	69 (60.5)	400 (80.2)	Reference
Preterm (< 37 weeks)	45 (39.5)	99 (19.8)	2.12 (1.53-2.94)
Rupture of ovular membranes			
Premature (preterm or term)	32 (28.1)	92 (18.4)	0.61 (0.42-0.89)
Other types	82 (71.9)	407 (81.6)	Reference
Meconium-stained amniotic fluid			
Yes	29 (25.4)	92 (18.4)	1.38 (0.95-2.01)
No	85 (74.6)	407 (81.6)	Reference
Labor dystocia			
Yes	6 (5.3)	133 (26.6)	0.18 (0.08-0.42)
No	108 (94.7)	366 (73.4)	Reference
Intrauterine resuscitation			
Yes	45 (39.5)	40 (8.0)	4.05 (3.00-5.45)
No	69 (60.5)	459 (92.0)	Reference
SGA			
Yes	57 (50.0)	122 (24.5)	2.42 (1.75-3.34)
No	57 (50.0)	377 (75.5)	Reference
Fetal gender			
Male	70 (61.4)	254 (50.9)	1.41 (1.00-1.99)
Female	44 (38.6)	245 (49.1)	Reference

**Table 2:** Univariate analysis of the associations of maternal, ultrasound, obstetric, and neonatal variables with a 5-min Apgar score < 7 and with the occurrence of an APO.

Variable	5-min Apgar score < 7		RR (CI 95%)	APO		RR (95% CI)
	Yes n (%)	No n (%)		Yes n (%)	No n (%)	
Maternal age (years)						
≤ 19	3 (12.5)	67 (11.4)	1.46 (0.42-5.05)	11 (11.6)	59 (11.4)	1.09 (0.60-1.97)
19-35	12 (50.0)	398 (67.6)	Reference	59 (62.1)	351 (67.8)	Reference
≥ 35	9 (37.5)	124 (21.0)	2.31 (0.99-5.36)	25 (26.3)	108 (20.8)	1.31 (0.85-2.00)
Skin color						
White	14 (58.3)	386 (65.5)	Reference	67 (70.5)	333 (64.3)	Reference
Non-White	10 (41.7)	203 (34.5)	0.74 (0.33-1.64)	28 (29.5)	185 (35.7)	1.27 (0.85-1.92)
Tobacco use						
Yes	4 (16.7)	65 (11.0)	1.58 (0.55-4.48)	7 (7.4)	62 (12.0)	0.63 (0.30-1.30)
No	20 (83.3)	524 (89.0)	Reference	88 (92.6)	456 (88.0)	Reference
Maternal disease						
Yes	20 (83.3)	456 (77.4)	1.43 (0.50-4.13)	72 (75.8)	404 (78.0)	0.90 (0.59-1.38)
No	4 (16.7)	133 (22.6)	Reference	23 (24.2)	114 (22.0)	Reference
Obesity						
Yes	12 (54.5)	307 (54.3)	1.00 (0.44-2.29)	42 (49.4)	277 (55.2)	0.82 (0.55-1.21)
No	10 (45.5)	258 (45.7)	Reference	43 (50.6)	225 (44.8)	Reference
Parity						
Primigravida	8 (33.3)	214 (36.3)	0.88 (0.38-2.02)	27 (28.4)	195 (37.6)	0.70 (0.46-1.06)
Multigravida	16 (66.7)	375 (63.7)	Reference	68 (71.6)	323 (62.4)	Reference
UA Doppler						
Normal	19 (79.2)	548 (93.0)	Reference	74 (77.9)	493 (95.2)	Reference
Abnormal	5 (20.8)	41 (7.0)	3.24 (1.27-8.29)	21 (22.1)	25 (4.8)	3.50 (2.39-5.11)
MCA Doppler						
Normal	15 (62.5)	482 (81.8)	Reference	46 (48.4)	451 (87.1)	Reference
Abnormal	9 (37.5)	107 (18.2)	2.57 (1.15-5.72)	49 (51.6)	67 (12.9)	4.56 (3.22-6.46)
CPR						
Normal	17 (70.8)	514 (87.3)	Reference	53 (55.8)	478 (92.3)	Reference
Abnormal	7 (29.2)	75 (12.7)	2.66 (1.14-6.23)	42 (44.2)	40 (7.7)	5.13 (3.68-7.15)
SDVP of amniotic fluid						
Normal	13 (54.2)	461 (78.3)	Reference	60 (63.2)	414 (79.9)	Reference
Abnormal	11 (45.8)	128 (21.7)	2.88 (1.32-6.30)	35 (36.8)	104 (20.1)	1.99 (1.37-2.88)
GA at delivery						
Term (≥ 37 weeks)	11 (45.8)	458 (76.8)	Reference	25 (26.3)	444 (85.7)	Reference
Preterm (< 37 weeks)	13 (54.2)	131 (22.2)	3.84 (1.76-8.40)	70 (73.3)	74 (14.3)	9.12 (6.01-13.83)
Rupture of ovular membranes						
Premature (preterm or term)	6 (25.0)	232 (39.4)	0.52 (0.21-1.30)	21 (22.1)	217 (41.9)	0.45 (0.28-0.70)
Other types	18 (75.0)	357 (60.6)	Reference	74 (77.9)	301 (58.1)	Reference
Meconium-stained amniotic fluid						
Yes	3 (12.5)	118 (20.0)	0.58 (0.18-1.92)	8 (8.4)	113 (21.8)	0.37 (0.19-0.75)
No	21 (87.5)	471 (80.0)	Reference	87 (91.6)	405 (78.2)	Reference
Dystocia						
Yes	4 (16.7)	135 (22.9)	0.68 (0.24-1.96)	9 (9.5)	130 (25.1)	0.36 (0.18-0.69)
No	20 (83.3)	454 (77.1)	Reference	86 (90.5)	388 (74.9)	Reference
Intrauterine resuscitation						
Yes	4 (16.7)	81 (13.8)	1.24 (0.43-3.54)	13 (13.7)	72 (13.9)	0.98 (0.57-1.69)
No	20 (83.3)	508 (86.2)	Reference	82 (86.3)	446 (86.1)	Reference
Mode of delivery						
Cesarean section	17 (70.8)	243 (41.3)	3.29 (1.39-7.83)	71 (74.7)	189 (36.5)	4.02 (2.60-6.20)
Vaginal	7 (29.2)	346 (58.7)	Reference	24 (25.3)	329 (63.5)	Reference
Cesarean section due to IFC						
Yes	5 (20.8)	109 (18.5)	1.15 (0.44-3.02)	28 (29.5)	86 (16.6)	1.83 (1.24-2.70)
No	19 (79.2)	480 (81.5)	Reference	67 (70.5)	432 (83.4)	Reference
SGA						
Yes	11 (45.8)	168 (28.5)	2.05 (0.94-4.49)	54 (56.8)	125 (24.1)	3.19 (2.21-4.61)
No	13 (54.2)	421 (71.5)	Reference	41 (43.2)	393 (75.9)	Reference
Fetal gender						
Male	12 (50.0)	312 (53.0)	0.89 (0.41-1.95)	47 (49.5)	277 (53.5)	0.87 (0.60-1.26)
Female	12 (50.0)	277 (47.0)	Reference	48 (50.5)	241 (46.5)	Reference

In the multivariate analysis, an abnormal UA RI and the need for intrauterine resuscitation retained their significance as risk factors for cesarean section due to IFC (RR = 2.03, 95% CI: 1.04-3.93, *p* = 0.03; and RR = 3.77, 95% CI: 2.53-5.62, *p* < 0.0001, respectively). For a 5-min Apgar score < 7, the risk factors were oligohydramnios (RR = 2.43, 95% CI: 1.07-5.54, *p* = 0.03) and prematurity (RR = 2.61, 95% CI: 1.04-6.86, *p* = 0.04). The variables considered significant risk factors for an APO were oligohydramnios (RR = 1.63, 95% CI: 1.04-2.54, *p* = 0.03), prematurity (RR = 5.15, 95% CI 2.94-9.03, *p* < 0.0001), and cesarean section (RR = 2.19, 95% CI: 1.14-4.20, *p* = 0.02). The EFW was not found to affect those results.

The most relevant predictor of cesarean section due to IFC demonstrated by the conditional inference tree model was the need for intrauterine resuscitation, regardless of the GA. A CPR ≤ 0.98 was also a significant predictor of that outcome. For a CPR > 0.98, the prevalence of cesarean section due to IFC was 10%, compared with 50% for a CPR ≤ 0.98. When the ultrasound variables were considered qualitatively normal or abnormal according to GA, the need for intrauterine resuscitation remained a relevant predictor of cesarean section due to IFC. However, a UA RI > the 95th percentile was also identified as a significant predictor of that outcome (cesarean section rate: 55% vs. 10%). For a 5-min Apgar score < 7, the most relevant predictor was low GA at delivery. A 5-min Apgar score < 7 was seen in 45% of the newborns at a GA < 29 weeks. It is essential to highlight that in that group (n = 26), the pregnancies ended in labor induction in four cases, spontaneous labor in four, and elective cesarean section due to chronic maternal disease in 19. However, for newborns at a GA ≥ 29 weeks, the most significant predictor of a 5-min Apgar score < 7 was a UA RI > 0.84. In the group with a fetal UA RI < 0.84 before delivery, the prevalence of that outcome was near zero.

The conditional inference tree model showed that a GA < 34 weeks at delivery was the most significant predictor of an APO, which occurred in 85% of the newborns at a GA < 34 weeks, compared with 30% of those born at a GA of 34-36 weeks and 5% of those born at a GA > 36 weeks. The prevalence of an APO was 25% among the newborns at a GA > 34 weeks with an abnormal CPR. Among the newborns with a normal CPR, oligohydramnios was a relevant predictor of an APO, which occurred in 12% of those with oligohydramnios, compared with 2% of those with normal amniotic fluid volume. Among the newborns at a GA > 37 weeks, the prevalence of an APO was near zero. Two other factors were significant predictors of an APO among the newborns at a GA < 37 weeks: an MCA RI < the 5th percentile; and cesarean section due to IFC. Among the preterm newborns with the brain sparing effect, the prevalence of an APO was 75% for those delivered by cesarean section, compared with only 25% for those delivered vaginally. Among the preterm newborns without the brain sparing effect, the prevalence of an APO was 30% regardless of the mode of delivery.

In the ROC curve analysis, all of the ultrasound variables presented poor performance in the prediction of cesarean section due to IFC and of a 5-min Apgar score < 7. However, an abnormal UA RI and an abnormal CPR presented moderate accuracy in predicting an APO, with AUCs of 0.76 (95% CI: 0.69-0.81) and 0.72 (95% CI: 0.65-0.77), respectively, whereas the other ultrasound variables all performed poorly in the prediction of an APO.

## DISCUSSION

Our study was motivated by recent publications that showed an association between an abnormally reduced CPR and the occurrence of APOs^(3,21-23,36,37)^. In the present study, a CPR ≤ 0.98 proved to be a significant predictor of cesarean section due to IFC, regardless of the GA at delivery, and a CPR < 1.0 proved to be a significant predictor of APO in newborns at > 34 weeks. Our results also show that maternal characteristics were not predictors of any APO. In a prospective study of women who gave birth to full-term fetuses, Fiolna et al.^(38)^ demonstrated that combining maternal characteristics with GA increased the accuracy of the prediction of cesarean section due to IFC. However, the addition of the CPR did not improve that accuracy. However, the features of our sample and the GAs at delivery are very different from those of the Fiolna et al.^(38)^ sample.

Despite the potential associations of ultrasound variables with cesarean section due to IFC, only an abnormal UA RI remained a significant risk factor in the multivariate analysis, cesarean section due to IFC having occurred in 55% of the cases in which the UA RI was abnormal, compared with only 10% of those in which it was normal. That is in agreement with the findings of DeVore^(3)^, Valiño et al.^(39)^, and Stumpfe et al.^(40)^ in samples of FGR fetuses. However, it differs from those of Vollgraff Heidweiller-Schreurs et al.^(41)^, who demonstrated the superiority of the CPR over UA Doppler in predicting cesarean section due to IFC. However, as previously mentioned, a CPR < 0.98 might also be a relevant predictor of that outcome, as demonstrated by other authors^(23,24,42)^. Nevertheless, it is crucial to highlight the differences among the studies. In addition, our study has a major limitation, which is the absence of pH results confirming neonatal acidosis. Furthermore, in the conditional inference tree model in which abnormal UA was shown to be a better predictor than the CPR, the ultrasound parameters were qualitative. Therefore, the use of a CPR cutoff of 0.98 could have influenced the findings.

Although the fact that the need for intrauterine resuscitation was an important predictor of cesarean section due to IFC seems like an obvious finding, only 55% of the cases in which intrauterine resuscitation was performed ended in cesarean section because the FHR pattern of IFC persisted thereafter^(6,43)^. However, this finding underscores the importance of the CPR as a predictor of the outcome, given that many of the pregnant women in whom IFC was suspected had previously been pregnant with a fetus with an abnormal CPR.

There were difficulties in comparing our findings related to a 5-min Apgar score < 7 with those of other studies because, in many of those studies, that outcome was included as composite neonatal morbidity, APO, or a similar term. In addition, our sample of pregnant women was quite diverse, with fetuses of various GAs and growth patterns. The variables considered risk factors for a 5-min Apgar score < 7 were oligohydramnios and a GA < 37 weeks at delivery. The latter was a relevant predictor of that outcome, which occurred in 45% of the newborns at a GA < 29 weeks, regardless of the ultrasound findings. However, among the newborns at a GA ≥ 29 weeks, the GA seemed to have a lesser effect and a UA RI > 0.84 was a better predictor of a 5-min Apgar score < 7. Therefore, GA at delivery was the factor that most influenced the neonatal outcome, especially when it was < 29 weeks, as has also been shown in other high-quality studies^(13,44)^. Oligohydramnios was a risk factor for a 5-min Apgar score < 7 but was not found to be a significant predictor of that outcome in the conditional inference tree model. The comparison of these findings with those of other studies was limited, because many authors have evaluated the association between the amniotic fluid index and APOs considering only term or post-term pregnancies.

A GA < 37 weeks at delivery was confirmed as the most critical predictor of an APO, especially for the newborns at a GA < 34 weeks, 85% of whom experienced an APO, compared with 30% and 5% for those born at 34-36 and > 36 weeks, respectively. As previously discussed^(13,44)^, prematurity might be predictive of an APO because of fetal organ immaturity. After week 34 of gestation, the gain in survival and reduction in neonatal morbidity is minimal^(45)^. Among all preterm newborns (GA < 37 weeks) in the present study, the brain sparing effect (MCA RI < the 5th percentile) was a predictor of an APO and acquired greater relevance when the newborn was delivered by cesarean section. Although this finding seems inconsistent, it should be borne in mind that the brain sparing effect is, initially, a protective event in a fetus with hypoxemia^(15)^. Fetuses with cerebral vasodilation are at lower risk of an APO in the early stages of hypoxia. In addition, our findings indicate that, for fetuses with the brain sparing effect, the risks of performing a vaginal delivery are outweighed by the benefits of that type of delivery in comparison with cesarean section^(46)^.

In our analysis of the newborns at a GA > 34 weeks, an abnormal CPR was associated with the occurrence of an APO, as demonstrated in previous studies^(3,24,41,47-49)^. However, there were major limitations to comparing our results with those of other studies of the topic. In many such studies, the reference values and CPR cutoff points were unclear, the GA limits were not stated, the features of the women and their pregnancies were not correctly presented, and the number of cases was insufficient to analyze the outcomes^(50)^. One possible explanation for our finding is the fact that the brains of late preterm and term fetuses are more sensitive to hypoxia than are those of preterm fetuses^(15)^. In a study of newborns at a GA of 35-37 weeks, Akolekar et al.^(49)^ found an association between the CPR and the occurrence of an APO, although they showed that the CPR had low accuracy and recommended caution in its use to predict that outcome.

Finally, as shown in our ROC curve analysis of the predictive power of the ultrasound variables (UA RI, MCA RI, CPR, and SDVP), the UA RI and CPR presented moderate accuracy in predicting the occurrence of an APO. That is in contrast with the findings that have been presented and discussed in other studies^(51)^.

In summary, GA is still a relevant variable that should be considered when proposing a delivery modality. An abnormal UA RI is a significant predictor of APO, especially in third-trimester, high-risk pregnancies. The CPR has emerged as a possible tool for predicting an APO, and, in our sample, it was found to be a predictor of cesarean section due to IFC and of an APO in late preterm and term pregnancies. However, when evaluated separately, the CPR presented moderate accuracy in predicting an APO. Therefore, its introduction into clinical practice should still be evaluated with caution.
